# Supplementary material for: DomiRank Centrality reveals structural fragility of complex networks via node dominance
Source: Nat Commun. 2024 Jan 2;15:56. doi: 10.1038/s41467-023-44257-0 (PMC10761873; doi:10.1038/s41467-023-44257-0)
Supplement: Supplementary file 1 — Supplementary Information [file 41467_2023_44257_MOESM1_ESM.pdf]

Supplementary Material: DomiRank Centrality reveals structural fragility of complex networks via node dominance

Marcus Engsig,[1,*] Alejandro Tejedor,[2,3,4,†] Yamir Moreno,[2,3,5] Efi Foufoula-Georgiou,[4,6] and Chaouki Kasmi[1]

[1]*Directed Energy Research Centre, Technology Innovation Institute, Abu Dhabi, United Arab Emirates*

[2]*Institute for Biocomputation and Physics of Complex Systems (BIFI), Universidad de Zaragoza, 50018 Zaragoza, Spain*

[3]*Department of Theoretical Physics, University of Zaragoza, Zaragoza 50009, Spain*

[4]*Department of Civil and Environmental Engineering, University of California Irvine, Irvine, CA 92697, USA*

[5]*CENTAI Institute, Turin 10138, Italy.*

[6]*Department of Earth System Science, University of California Irvine, Irvine, CA, USA*

(Dated: December 5, 2023)

CONTENTS

I. Understanding power shifts in Rich-Clubs using DomiRank 3

II. DomiRank and strict symmetry constraints: even Lattice 5

III. Finding Optimal σ 6

IV. Correlations of DomiRank with other centralities 9

V. Heterogeneous networks 11

VI. Collective Influence (CI) and DomiRank 14

A. Toy Networks 15

VII. Link removal during attacks 19

VIII. Networks undergoing random-recovery 19

References 23

References 23

* marcus.w.engsig@gmail.com

† alej.tejedor@gmail.com

I. UNDERSTANDING POWER SHIFTS IN RICH-CLUBS USING DOMIRANK

In this section, we present a first exploration of Rich-Club networks via DomiRank to provide insight into (i) the joint dominance (collusion) mechanism that emerges from competition dynamics and (ii) how changing the connectivity of the Rich Club nodes and/or their peripheries alters in an interpretable manner the relative dominance of Rich-Club's individuals and therefore, the overall power dynamics of Rich-Club networks.

Rich-Club networks are important for understanding various types of real networks - i.e., political networks, macroscale brain networks, and many more [1–4]. For these networks, commonly used centralities have difficulty distinguishing the importance between nodes within a Rich Club, as their nodes establish a closely related 'hierarchy' of hubs. Here, DomiRank offers a novel insight to reveal key properties of the structure and dynamics of Rich-Club networks. For this section, we explore a simple version of a Rich-Club network, which consists of an interconnected Rich Club of hub nodes. Each hub node in the Rich Club is a star network with a varying number or nodes attached to them (periphery). Note that we keep the total size of the network sufficiently small to enhance interpretability and visually guide the understanding of the underlying dynamics in terms of DomiRank.

Fig. S1 illustrates how DomiRank centrality can be instrumental in providing fundamental understanding of the power dynamics on Rich-Club networks characterized by highly competitive relations. Fig. S1a displays the chosen initial configuration of the Rich-Club, where node coloring represents nodal dominance given by DomiRank centrality ($\sigma = \frac{0.99}{-\lambda_N}$), and nodes are arbitrarily labeled with numbers to ease the discussion that follows. Here, node 1 is dominating (highest DomiRank score) as it has the largest periphery, and additionally, it is jointly dominating nodes 2 and 6, by colluding with nodes 3 and 5, respectively. Thus, nodes 3 and 5 exhibit dominant behavior, although below the levels obtained by node 1. Actually, node 1 can reinforce its dominant role by establishing direct competition (links) with nodes 3 and 5 (Fig. S1b). In such a scenario, node 1 is able to subdue nodes 3 and 5 with the help of node 4, which contributes via joint dominance. A more drastic power shift such that nodes 3 and 5 jointly dominate the rich club can also be induced by establishing competition between nodes 5 and 2, and 3 and 6 (Fig. S1c,d respectively).

Furthermore, changes in the relative dominance of the nodes in the rich club can be

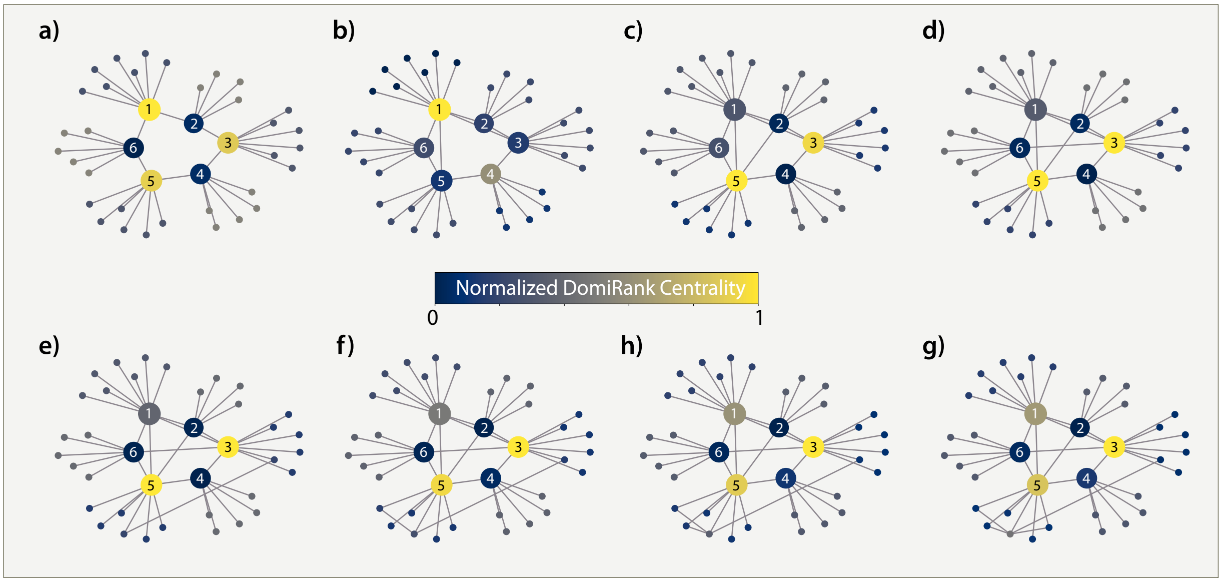

FIG. S1. **Steering dominance in a Rich-Club network by altering connectivity**. The different panels show changes in dominance (color-encoded) starting from a (a) simple Rich-Club network when new competition (links) is established within the Rich-Club and/or in its periphery (note that some nodes are arbitrarily labeled for reference). In particular, the following new links were introduced in each of the panels: (b) link (1,3) and link (1,5); (c) link (2,5); (d) link (3,6); (e) link between the periphery of node 3 and the periphery of node 5; and (f,g,h) internal competition (addition of links) in the periphery of node 5. In all the panels, the DomiRank centralities are computed for $\sigma = \frac{0.99}{-\lambda_N}$ in order to simulate a highly competitive environment where changes in power dynamics are more likely to occur.

promoted by modifying the connectivity of their peripheries. Thus, the dominance of node 3 over node 5 can be induced by creating competition in the periphery of node 5, which would internally challenge the dominance of node 5 (Fig. S1e-g). In other words, an increase in the connectivity (competition) of the periphery reduces the relative dominance of the center node connected to that periphery (node 5), making its periphery less dominated and more 'fit'. The power shift observed from introducing competition in the periphery of a hub is not as drastic as that of creating direct competition for members within the Rich Club; however, it is sufficient to steer the power balance within the Rich Club.

These simple examples show how DomiRank is able to naturally describe collusion scenarios where nodes can dominate neighborhoods and gain power by establishing common

enemies. Therefore, we show that we can steer power dynamics by artificially forcing competition and joint dominance between Rich-Club nodes or by encouraging competition within a hub periphery.

II. DOMIRANK AND STRICT SYMMETRY CONSTRAINTS: EVEN LATTICE

As demonstrated by the results shown in the main text, DomiRank is able to integrate, for large values of σ, global (meso- to large-scale) network properties. This is particularly apparent in the case of a square lattice network with an odd number of nodes (see Fig. 2 in the main text), where the entire network properties partially influence the value of each node's DomiRank via the competition mechanism. At the limit of high σ an alternating spatial pattern emerges which reflects two global network properties: the finite boundary and global symmetries.

However, in rare cases, strict symmetries can exert constraints that hinder the emergence of a fully developed dominance pattern. A prime example of such a scenario corresponds to a square lattice with an even number of nodes (see Fig. S2a). We show that in such a network, due to the degree homogeneity and strict symmetry, DomiRank cannot establish an alternating pattern of dominating-dominated nodes similar to the one exhibited by the square lattice with an odd number of nodes, as it would break the system's symmetries. Thus, for instance, the spatial pattern of DomiRank obtained for a square lattice of 64 nodes is shown in Fig. S2a at the limit $\sigma \rightarrow \frac{1}{-\lambda_N}$. We note that for the DomiRank distribution based on this value of σ, attacks are still more efficient than those based on different centralities (see Fig. S2b). However, the overall efficiency is significantly lower than for the DomiRank-based attack for a square lattice with an odd number of nodes, as its superior efficiency relies on the alternating nature of the spatial distribution. Interestingly, we have found numerically that in the case of the lattice with an even number of nodes, the optimal value of the parameter corresponds to $\sigma_{opt} = \frac{1.25}{-\lambda_N}$, i.e., a case of supercharged competition. Note that for this network, despite σ being outside of the interval $\left(0, \frac{1}{-\lambda_N}\right)$, the recursive formula still yields numerical convergence (to tolerance levels of at least 10^{-6}). For this state of supercharged competition, an alternating spatial pattern of DomiRank is obtained (Fig. S2c), which, although different from that displayed for the lattice with an odd number of nodes, is able to produce comparably efficient attacks with the odd case

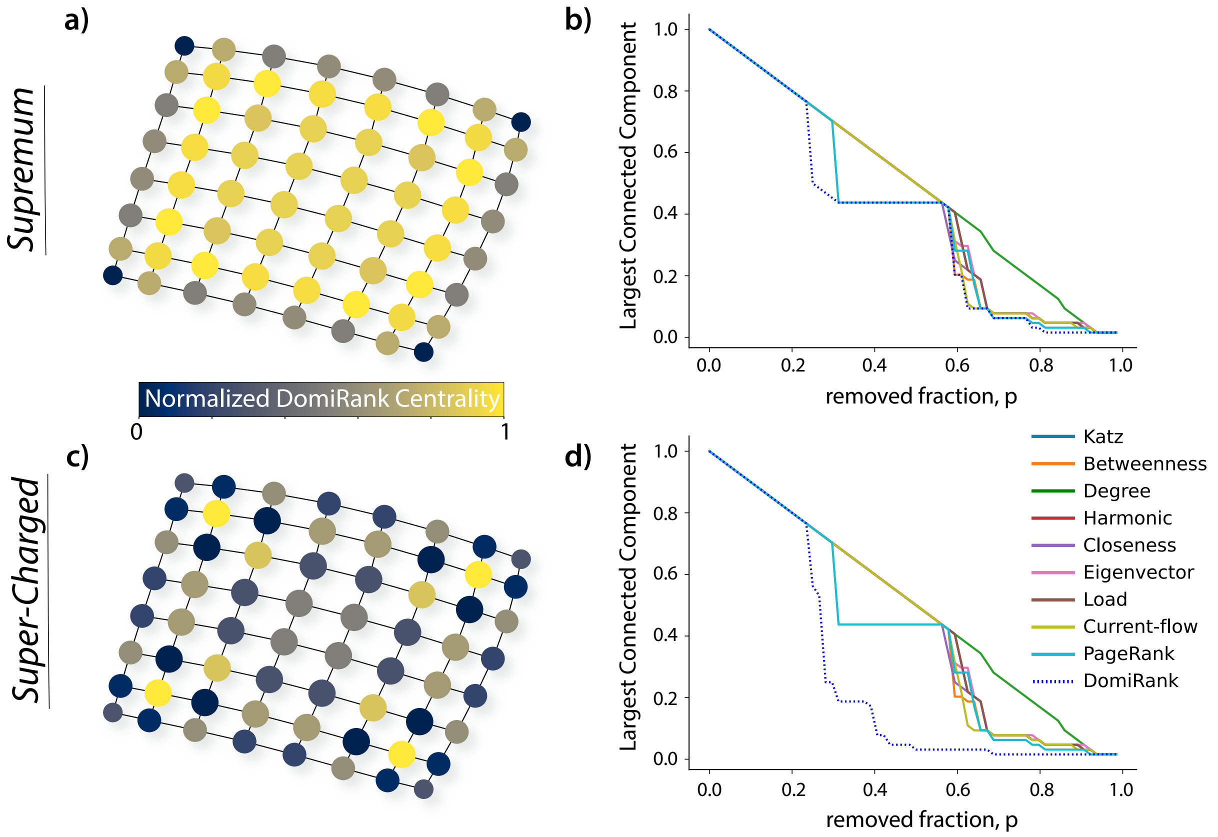

FIG. S2. **Supercharging DomiRank competition on an even lattice**. DomiRank centrality distributions in an even lattice of size $N = 8\times 8$ for competition levels corresponding to (a) $\sigma = \frac{0.99}{-\lambda_N}$ and (c) $\sigma = \frac{1.25}{-\lambda_N}$. Panels (b,d) show the deterioration in the relative size of the largest connected component according to the attack strategies based on the centrality values shown in panels (a,c), respectively, along with nine other centralitiy-based attacks.

(Fig. S2d). When a similar supercharged competition was explored in other topologies, the analytical DomiRank calculation led to suboptimal attack strategies, and numerical divergence via the recurrence equation was obtained.

III. FINDING OPTIMAL σ

DomiRank has a tuneable parameter σ, that allows modulation of the level of competition introduced in the dynamical equation. In order to find the optimal σ, we first define our loss function - the area under the largest-connected-component curve generated from a network undergoing sequential node removal - and thereafter numerically explore the loss function

for the full range of σ values. Choosing the σ that corresponds to the smallest loss, yields the optimal σ^* in terms of efficiently dismantling the network.

In this section, we show the loss curves for varying σ for various topologies - both real and synthetic. The loss curves are displayed for two main reasons: (i) to show the typical behavior that they exhibit for different topologies, and (ii) to demonstrate that this methodology is applicable to massive networks, as the space can be searched for a network size of nearly 24 million nodes.

In Fig. S3, we show how some demonstrative loss curves look for different synthetic topologies and for a real-world network (the full US roads network) consisting of $N = 23,947,347$ nodes. The optimal level of competition (σ^*) is displayed in each panel. We want to highlight a few important observations from Fig. S3: (i) Loss curves exhibit clear decreasing and/or increasing trends towards the area of optimal σ (with some variability); (ii) For many topologies, there is a range of values of σ for which the attack is near optimal; And (iii) different topologies require a different trade-off of local vs. global information (σ) in order to dismantle the network most efficiently. Finally, Fig. S3d shows that the optimal parameter can also be found for massive networks.

Regarding the computational cost of exploring the optimal value of σ, we first need to consider that the cost of computing the connected components of a graph scales with $\mathcal{O}(N + m)$ [5]. Secondly, we can recompute the largest-connected-component size not after every node removal but sample the curve at a given frequency - e.g. set it to be recomputed every 1% of nodes removed. Therefore, if we assume that links are removed linearly (which is near worst case - see Fig. S10), then the computational cost for dismantling the entire network and recomputing the largest connected component every 1% (100 times) scales with $\mathcal{O}((\frac{100+1}{2}(N + m))$. Finally, computing the area under the largest connected component for different σ are independent calculations, and thus, this loss function as a function of σ can be parallelized, and sped up almost linearly with the number of threads.

Note that other centrality metrics also have a parameter in their definition. In those cases, we have used their standard values for our analysis. Thus, Katz's and PageRank's parameters were chosen to be 0.01 and 0.85, respectively. The Katz's parameter was reduced to 0.001 occasionally, when convergence was not found within 1000 iterations. We have further tested that this election is not detrimental to their performance in comparison to DomiRank and other metrics. Fig. S4 shows that the gain from choosing the optimal value

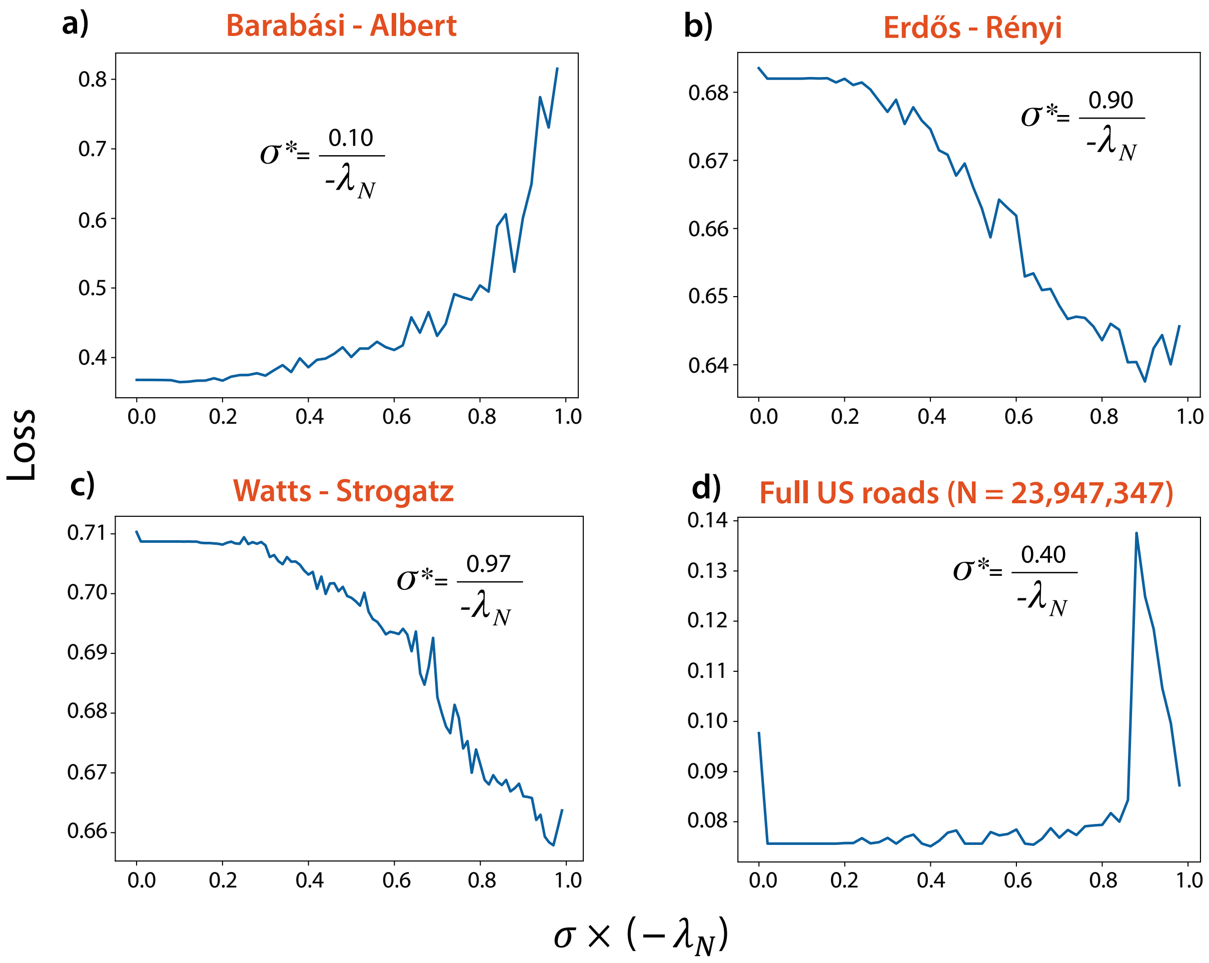

FIG. S3. **DomiRank's loss functions for different topologies.** The DomiRank's loss functions computed for the complete range of the free parameter (σ) are shown for three different synthetic networks of size $N = 1000$: (a) Barábasi-Albert, (b) Watts-Strogatz, and (c) Erdős-Rényi, and for (d) a massive real network, the Full US Road network, consisting of nearly 24 million nodes. The optimal values of σ^* corresponding to the locations of the loss function's global minima are displayed for each panel. Note that all the curves shown were computed using 100 samples linearly distributed in the interval $(0, \frac{1}{-\lambda_N})$.

for the Katz and PageRank parameters is marginal when compared to their default values. Finally, we also show that DomiRank tends to outperform the other metrics, regardless of the σ parameter.

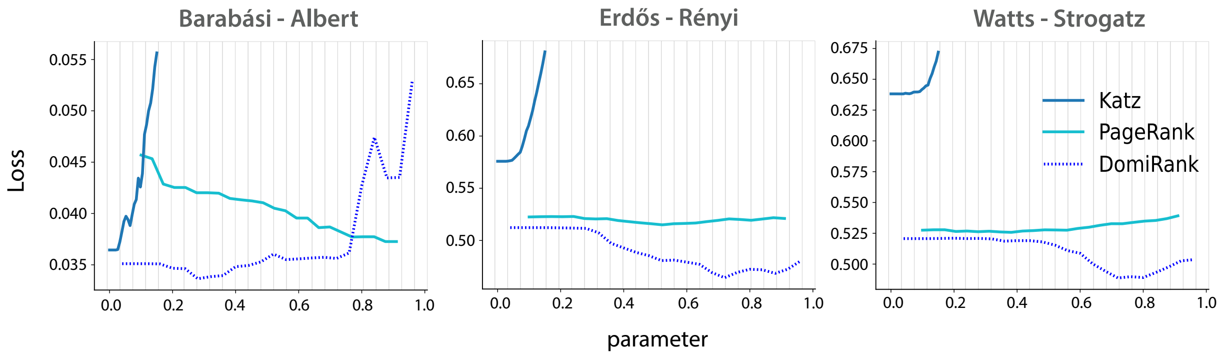

FIG. S4. **Comparing the loss functions of PageRank, Katz, and DomiRank within their parameter space.** The loss functions corresponding to PageRank, Katz, and DomiRank computed for the complete range of their free parameters are shown for three different synthetic networks of size $N = 300$, namely, (a) Barábasi-Albert, (b) Erdős-Rényi, and (c) Watts-Strogatz. Note that all the curves shown were computed using 25 samples linearly distributed in their respective parameter domains.

IV. CORRELATIONS OF DOMIRANK WITH OTHER CENTRALITIES

Here, we look at various pairwise Spearman correlations between the centralities tested on four synthetic topologies, namely, (i) Barábasi-Albert, (ii) Erdős-Rényi, (iii) Random Geometric Graph, (iv) and a 2D lattice, in order to gain insights into how DomiRank dismantles a network.

The DomiRank centrality is derived as the steady-state solution of a competition-based dynamical equation, yielding nodal scores inherently different from previous methods. The Spearman correlation provides a good tool to understand how similar DomiRank is to other previous centrality methods. Conceptually, we know from the dynamical equation that as $\sigma \to 0$, DomiRank, by definition converges to the degree distribution, and as $\sigma \to \frac{1}{-\lambda_N}$ an increasing amount of competition (mesoscale and global information) is considered in the dynamics and that adjacent nodes tend to have disparate scores, which, is a quite distinctive signature of DomiRank with regards to other centrality measures.

Fig. S5(a-d) presents the pairwise Spearman correlation for several centralities included in our analysis. As noted before, the optimal value of σ depends on the topology considered.

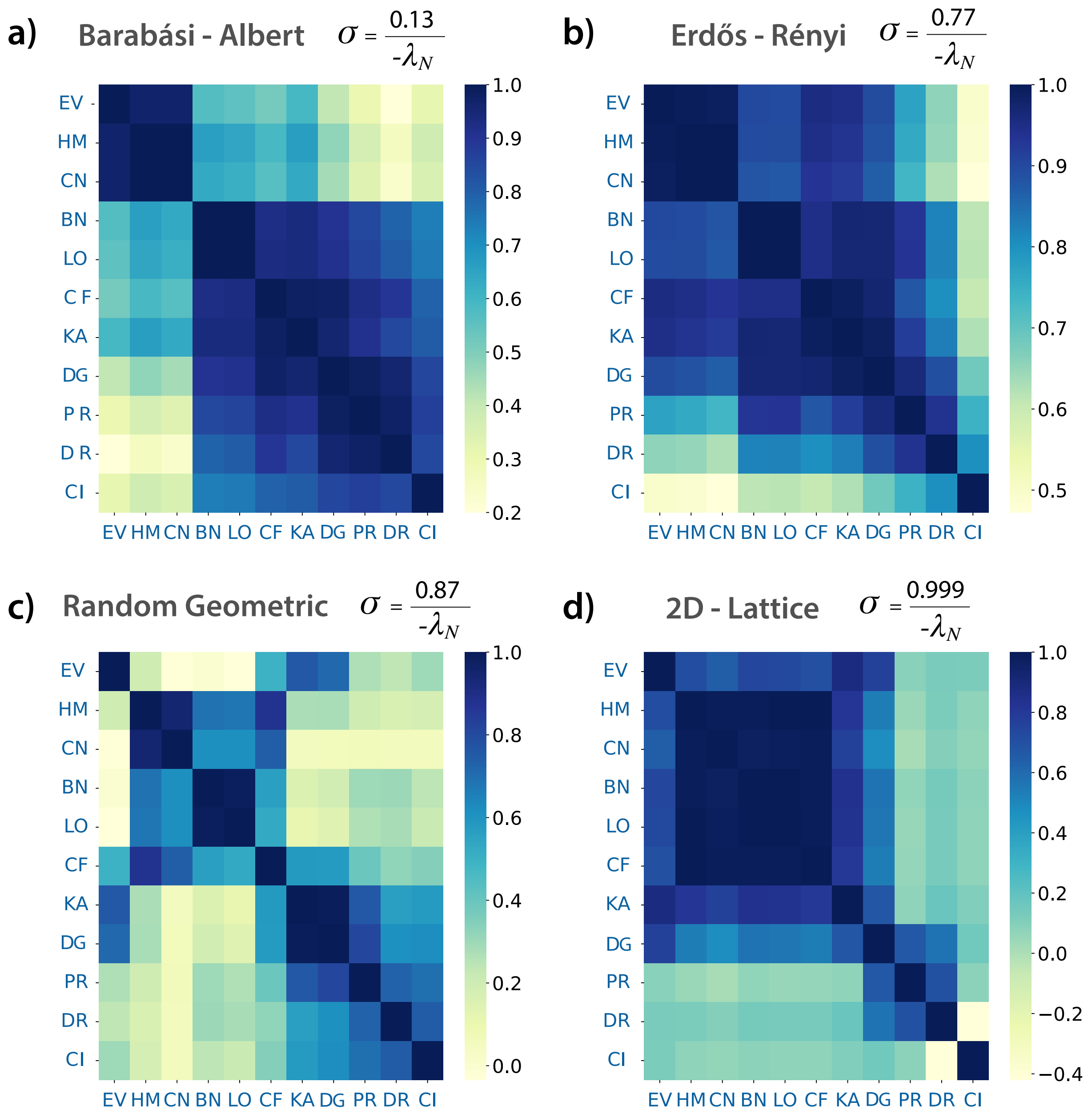

FIG. S5. **Pairwise Spearman correlation between different centralities.** The pairwise Spearman correlation matrices for the different centralities used in our study are displayed as heatmaps for four different topologies, namely, (a) Barábasi Albert ($N = 1000$), (b) Erdős-Rényi ($N = 1000$), (c) Random Geometric Graph ($N = 1000$), and a regular 2D-Lattice ($N = 33 \times 33$). Particularly, the centralities compared are Eigenvector (EV), Harmonic (HM), Closeness (CN), Betweenness (BN), Load (LO), Current-Flow (CF), Katz (KA), Degree (DG), PageRank (PR), DomiRank (DR), and Collective Influence (CI).

The topologies analyzed allow us to explore a range of σ increasing progressively from Barábasi-Albert to the 2D-lattice (Fig. S5(a-d)). Within the gradient of σ, we observe the expected progressive divergence of DomiRank from degree centrality. We also observe that DomiRank displays different degrees of correlation to different centralities depending on the topology examined, providing further evidence of the adaptability of DomiRank by varying the value of σ to mine the most relevant structural features of each topology.

For its significance, we have also included a comparison of DomiRank with Collective Influence (CI) [6], despite the latter being the only centrality in this analysis relying on an iterative computation (more details see section S-VI). Interestingly, DomiRank offers high values of Spearman correlation with CI, particularly as σ increases (see Fig. S5a-c). This observation is compatible with the fact that both metrics can extract global information to inform the individual nodal centrality and avoid artificially high values for neighboring nodes (although through very different mechanisms - see section VI for more details). It might seem striking at first glance that for the case of the 2D-lattice, the Spearman correlation between CI and DomiRank is the lowest among metrics despite the large value of the σ parameter in that case. However, this example should not be taken as evidence of the divergence of the two metrics for very large values of σ; actually, a closer examination of those two metrics on the 2D-Lattice (see Section S-VI) reveals that they differ in the spatial pattern due to the strict symmetry of the network, but they still exhibit similar strategies creating alternating patterns of high-low values.

V. HETEROGENEOUS NETWORKS

Figure 5 in the main text shows the evolution of the largest connected component for different synthetic and real-world networks as they are attacked based on various centrality metrics. DomiRank-based attacks outperform (with different degrees of significance) all other attacks for all the networks analyzed but in one case. That case corresponds to a massive social network (LiveJournal users and their connections - see Figure 4k in the main text), where the DomiRank-based attack, although very competitive, does not perform better than the PageRank-based attack.

In this section, we pose the hypothesis that the presence of heterogeneity (different structural properties) in different subgraphs of the network could lead to underperformance for

DomiRank-based attacks. The rationale behind this statement is straightforward in the light of previous results. As shown in the analysis of synthetic networks in Fig. 3 and Fig. 4 of the main text, DomiRank excels in highlighting the important nodes with different values of σ depending on the network structure. Thus, for hub-dominated networks, a relatively low value of σ provides the most effective ordering of nodes for designing attack strategies. On the other hand, more regular networks such as lattices or random graphs require large values of σ, which allow for a larger integration of the information in the network structure for assessing the relative importance of each node. Consequently, if a network consists of different subgraphs (e.g., communities) with different topological properties might require different σ for each subgraph since an *average* global value of σ would lead to suboptimal results.

In order to address this issue, we could substitute the σ parameter in DomiRank definition by a diagonal matrix (without additional computational cost), where the entries of $\sigma_{i,i}$ are corresponding to the optimal σ for the community that node i belongs to. We can mathematically describe this new diagonal matrix σ as follows, given T communities $C_j, j \in [1, T]$;

$$(\sigma)_{i,i} = \sum_{j=1}^{T} \sigma_j \mathbb{1}_{i \in C_j}. \tag{1}$$

Moreover, in order to guarantee the convergence of DomiRank, Eq. 1 takes the final form:

$$(\sigma)_{i,i} = \min \left[\sum_{j=1}^{T} \sigma_j \mathbb{1}_{i \in C_j}, \frac{-1}{\lambda_N} \right] \tag{2}$$

where λ_N is the minimum (largest negative) eigenvalue of the whole network.

To test this hypothesis, we generate synthetic networks consisting of two subgraphs, each of them generated with a different model (e.g., Barábasi-Albert and random geometric graph), establishing links between both subgraphs (10% of the nodes establish a connection with a node in the other subgraph). Fig. S6 displays the results for the attacks based on the previous centralities (including DomiRank), as well as the results obtained from a DomiRank where nodes in different subgraphs are evaluated with different values of σ to account for heterogeneity in networks. As expected, in the cases where the two merged networks are characterized by disparate values of σ for optimal attack strategies (i.e., large heterogeneity), we obtain a larger gain by considering the diagonal-matrix-based σ in the definition of DomiRank. Thus, for instance, Fig. S6a shows a significant improvement in

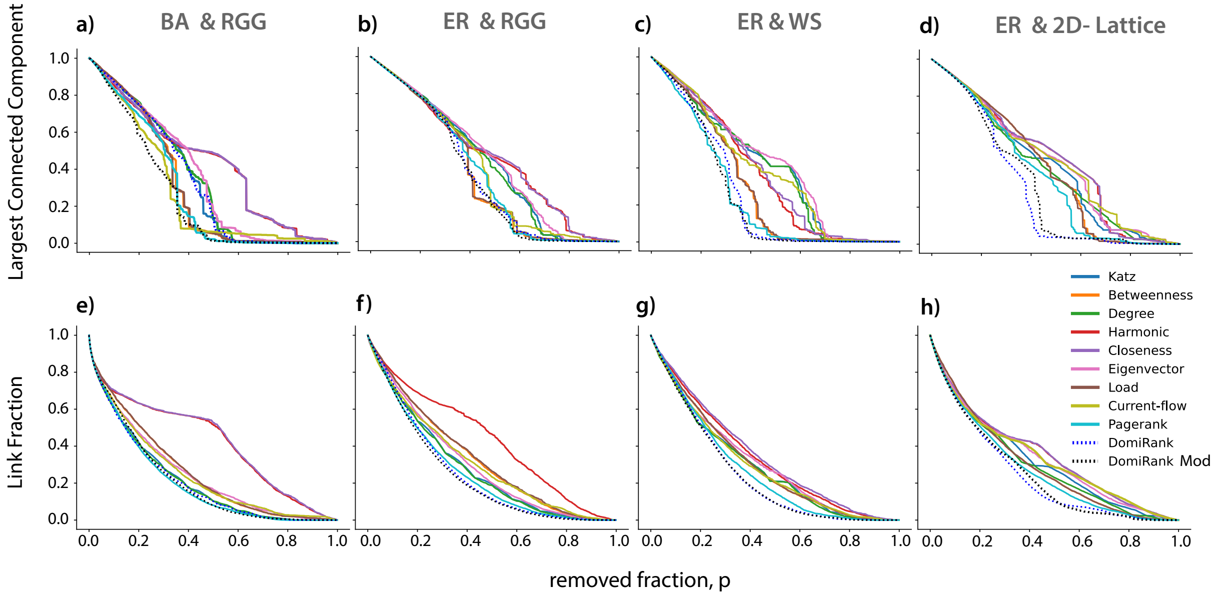

FIG. S6. **The effect of heterogeneity on the performance of centrality-based attacks on synthetic networks.** Panels a-d show the evolution of the relative size of the largest connected component and panels e-h show the evolution of the remaining link fraction whilst undergoing sequential node removal according to descending scores of various centrality measures for different coupled synthetic networks (heterogeneous) of size $N = 1000$: (a,e) Barábasi-Albert (BA) and random geometric graph (RGG), (b,f) Erdős-Rényi (ER) and random geometric graph (RGG), (c,g) Erdős-Rényi (ER) and Watts-Strogatz (WS), and (d,h) Erdős-Rényi (ER) and a 2D-lattice. Here we have two different DomiRank-based-attacks corresponding to two different σ (i) the optimal σ for the entire network (denoted in the legend as DomiRank), and (ii) the capped optimal diagonal matrix σ as per eq. 2 (denoted in the legend as DomiRank Mod).

the performance of the attack strategy when heterogeneity is considered in the definition of DomiRank for a network consisting in the combination of a subgraph generated by a Barábasi-Albert model with relatively low degree ($\bar{k} = 4$) and random-geometric-graph ($\bar{k} = 5$). Additionally, from Fig. S6c we see that by combining two subgraphs characterized by comparable optimal σ values like the Erdős-Rényi and Watts Strogatz networks, the gain is just incremental. Note that when the difference in the optimal value of σ does not significantly differ between the two subgraphs (e.g., Erdős-Rényi and 2D-Lattice - See fig. S6 d), the traditional DomiRank computed in the whole network might offer better

performance than the community-based version, as it accounts for the links connecting the two sub-graphs.

Consequently, massive networks consisting of multiple communities with different properties might require the adoption of the definition of DomiRank that accounts for that heterogeneity (i.e., using the diagonal-matrix-based σ) to design more efficient attack strategies for dismantling the networks, as the traditional definition of DomiRank could underperform.

Thus, by combining the various algorithms to detect communities in massive networks, and this newly defined σ could potentially lead to further gains in designing strategies to dismantle networks' structure and functionality without incurring unaffordable computational costs.

VI. COLLECTIVE INFLUENCE (CI) AND DOMIRANK

In this section, Collective Influence (CI) and DomiRank are compared side-by-side, to understand their differences in terms of (i) underlying principles and (ii) ability to dismantle toy networks.

CI, a relatively new centrality metric introduced by Morone and Makse [6], is defined for a node i as follows,

$$CI_\ell(i) = (k_i - 1) \sum_{j \in \partial B(i,\ell)} (k_j - 1), \tag{3}$$

where, ℓ is a parameter, $\partial B(i,\ell)$ is the frontier of the ball containing the nodes around node i at a distance ℓ (without back-propagation), and k_i, k_j are the degrees of node i, j respectively. CI has a low computational cost, but it is an iterative centrality: once the CI is computed for all the nodes in the network, the node with the highest CI value is formally assigned this value and removed from the network. Then, the CI is recomputed for all the remaining nodes, repeating the process of assignation and removal until all nodes are removed.

CI measures the multiplicative cascade of paths that each node could develop at various scales through a tuneable parameter. Morone and Makse [6] showed that collective influence found novel influencers in networks that had remained unseen by previous centrality measures [7]. These novel influencers were identified to be nodes surrounded by hierarchical coronas of hubs, making them potential epicenters for spreading processes. In our study, we

follow the methodology of Pei et al. [8], and set $\ell = 2$.

On the other hand, DomiRank identifies the relative dominance of the different nodes in their respective neighborhoods, capitalizing on a dynamical equation that incorporates a competition term and a relaxation term. For ease of comparison, DomiRank's underlying dynamical equation, described in detail in the main text, is displayed here:

$$\frac{d\Gamma_i(t)}{dt} = \sigma \sum_{j=1}^{N} [A_{ij}(1 - \Gamma_j(t))] - \Gamma_i(t), \tag{4}$$

where σ (tuneable parameter) modulates the relative extent of competition in the network and, thus, the amount of global information considered by DomiRank. Note that in this version of the equation, other parameters (β and θ) of the more general dynamical equation (see Eq. 1 in the main text) that do not impact the final relative value of the DomiRank distribution are considered equal to one for simplicity.

Both CI and DomiRank are able to provide global information that is not redundant in neighboring nodes. However, the ways they extract this global information and avoid artificial local correlations are entirely different. Thus, global information is integrated by DomiRank via a competition dynamic, while collective influence accounts for the multiplicative cascade of paths that each node could develop at various scales. In terms of avoiding spurious correlations among neighboring nodes, CI requires an iterative recomputation wherein the most influential node is iteratively removed between each computation of CI, whereas DomiRank is computed only once for the entire network, and it is its underlying dynamic process that is able to inherently provide centrality scores that are not locally redundant. In order to further understand the differences and similarities between DomiRank and CI, we compare the two centralities for small toy networks.

A. Toy Networks

A particularly insightful example for highlighting the differences between DomiRank and CI is the 2D lattice network, which has two key properties - (i) degree-homogeneity apart from the edge of the lattice, and (ii) ordered structure with high symmetry. Fig. S7a,b reveals that both centrality distributions exhibit alternating spatial patterns in the network domain. We want to highlight once more here that those alternating patterns emerging for both metrics respond to completely different mechanisms - while it is a direct consequence

of the dynamics of DomiRank for highly competitive environments, it is not intrinsic to the definition of CI (see S7c for the initial evaluation of CI for every node) but it is the effect of the iterative recomputation of CI after each node removal.

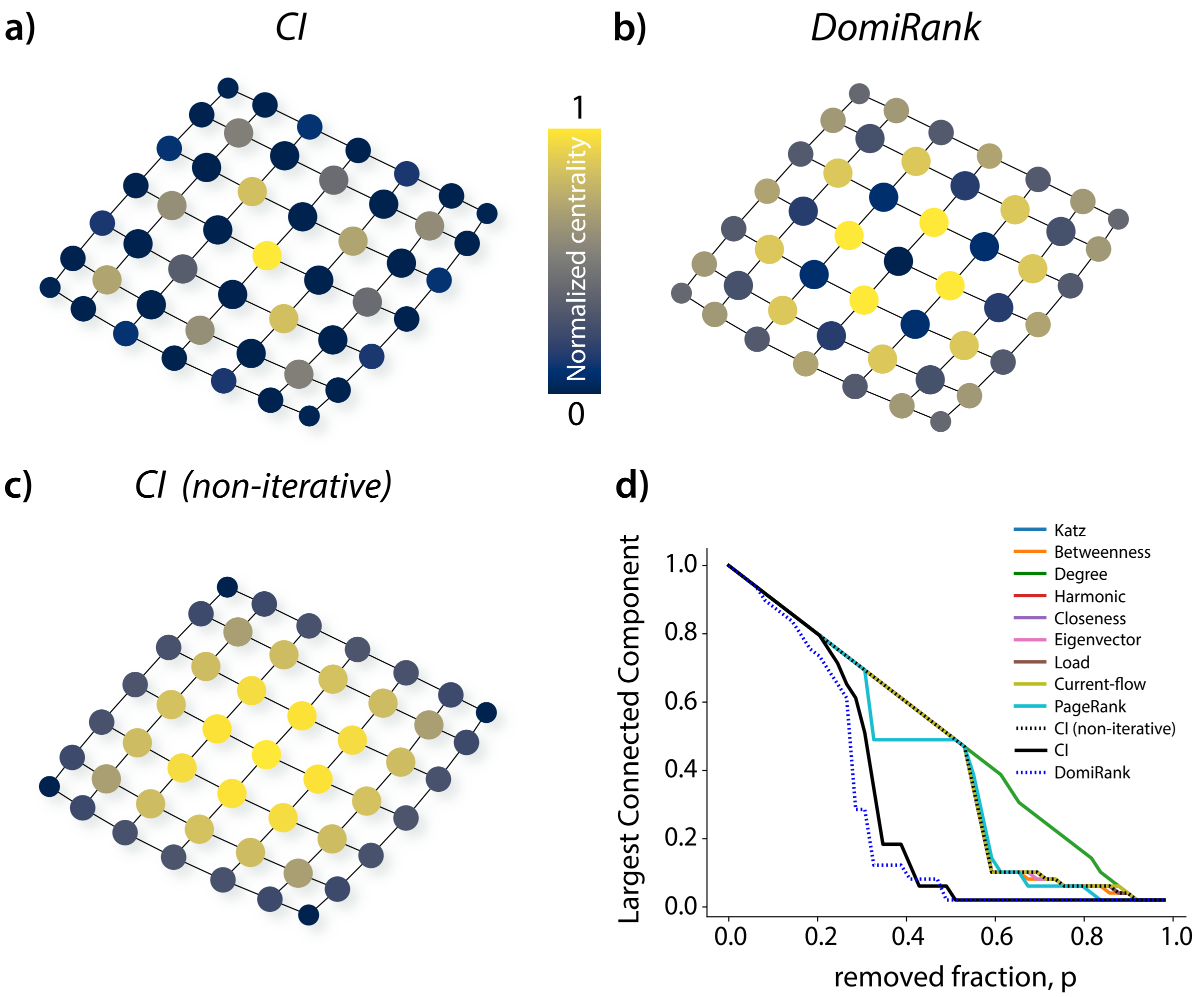

FIG. S7. **Collective Influence and DomiRank on a regular 2D lattice.** Comparison between three centralities on a 2D regular lattice ($N = 49$), namely, (a) Collective Influence, (b) DomiRank, and (c) non-iterative Collective Influence, along with (d) the evolution of the relative size of the largest connected component whilst undergoing sequential node removals according to the displayed centralities.

A closer look at Fig. S7a,b shows that the two spatial patterns are inverted with respect to each other (see also Fig. S5d for correlations). These antagonistic patterns could be distilled down to the identification of the most important node in the network (as this determines

the alternating spatial pattern given the network symmetry). CI finds that the central node is the most important node for a multiplicative cascade of paths, and DomiRank finds that the central node is the most dominated node through the collusion of its neighbors. DomiRank, rather than removing the central node, isolates it, impeding as well its potential in a spreading process whilst continuously damaging the network. Note that both strategies yield very efficient attack strategies, but the highlighted differences between DomiRank and CI lead to slightly better performance for DomiRank-based attacks, as shown by Fig. S7d.

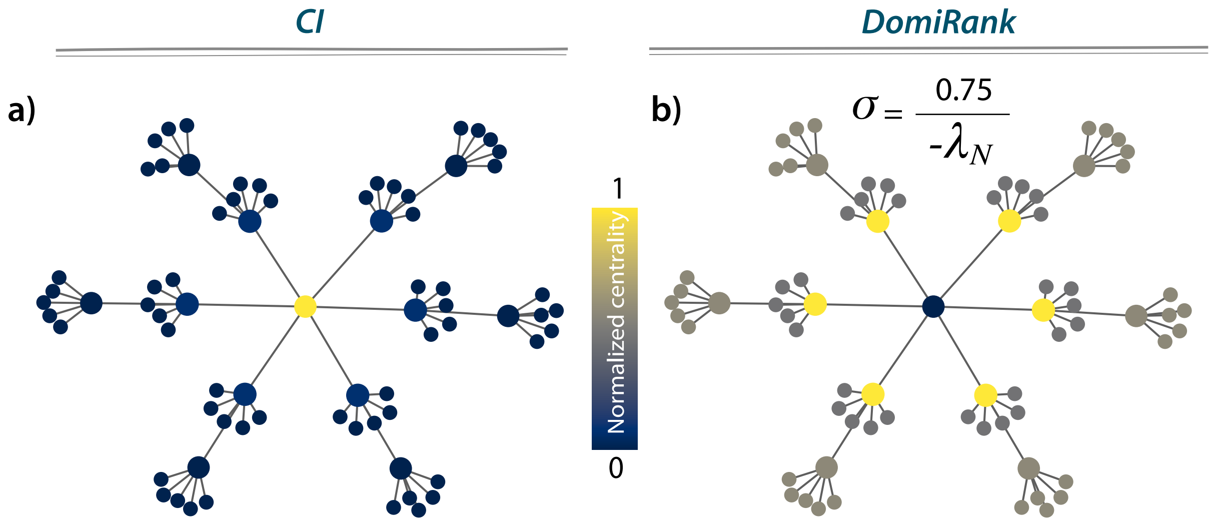

FIG. S8. **Comparison of Collective Influence and DomiRank on a network consisting of a corona of hubs.** A comparison between (a) Collective Influence and (b) DomiRank scores to highlight their differences in evaluating nodal importance. Note that DomiRank is computed and displayed for $\sigma = \frac{0.75}{-\lambda_N}$, but for this topology, a similar pattern of the relative ranking of nodes is obtained for most of the range of σ values.

Another illuminating network configuration to display the differences between CI and DomiRank is displayed in Fig. S8, where a corona of hubs is presented with the (a) CI and (b) DomiRank centralities displayed by the node coloring. This figure shows how CI is able to identify the central node as the most influential. From DomiRank's perspective, that same node is jointly dominated by its neighbors - i.e., the hierarchical corona of hubs. From a network-dismantling perspective, a DomiRank-based attack would sequentially remove the hubs surrounding the central node (similarly to the strategy followed for the 2D-Lattice), which satisfies two objectives: dismantling the hubs as fundamental parts of the structure

of the network and continuously decreasing the influence of the central node (top influencer from the CI perspective) by isolating it. On the other hand, a CI-based attack would first remove the central node and then proceed with the removal of the hubs, which remain important due to their high degree. The pragmatic strategy of DomiRank, wherein it attempts to remove important influencers whilst decreasing the importance of the top influencer, yields competitive, if not superior, attack strategies in dismantling networks despite of not being recomputed sequentially after each nodal removal.

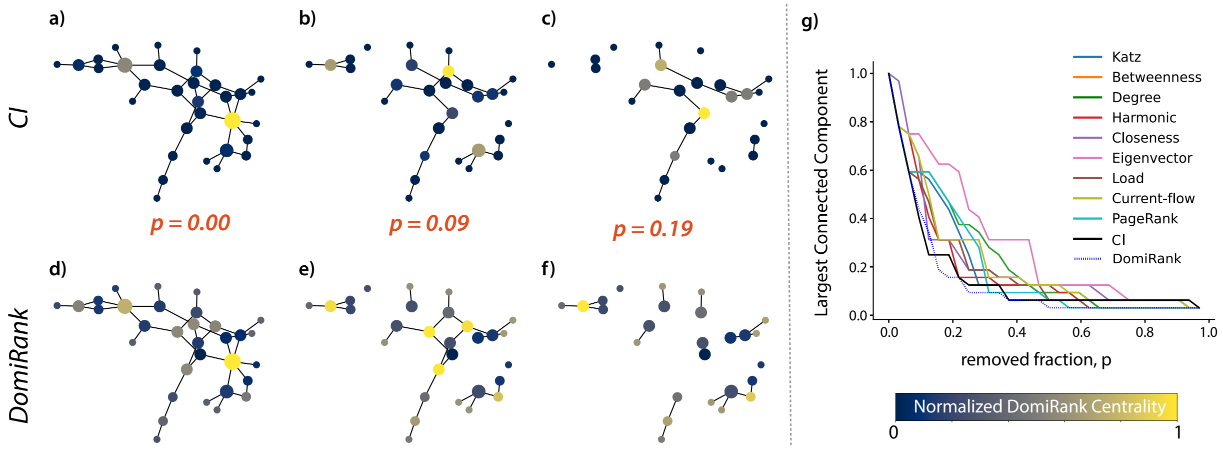

FIG. S9. **Comparison of Collective Influence and DomiRank on a toy random network.** Comparison between (a,b,c) Collective Influence and (d,e,f) DomiRank and how they dismantle a toy Erdős-Rényi network of size $N = 32$ when nodes are sequentially removed according to descending values of their centralities. (g) The evolutions of the largest connected component of the network undergoing CI- and DomiRank-based attacks are displayed, along with various attacks based on other centralities for reference. Note that the color scale used to represent the relative values of the centralities in panels (a-f) is re-normalized for each panel for visualization purposes

Yet another interesting example for the comparison of CI and DomiRank is the Erdős-Rényi network in Fig. S9, where a mix of local and mesoscopic features might emerge. Here, Fig. S9(a,d) shows that CI and DomiRank agree on the identification of the two most important nodes despite their underlying mechanistic differences. However, the relative importance of nodes differs for the other intermediate nodes. Particularly, Fig. S9(b,e) shows a similar phenomenon as highlighted by Figs. S7, S8, that the most important node, according to CI, is one of the least important nodes for DomiRank, as DomiRank aims to

isolate those nodes by removing their periphery. This effect is further shown in the temporal evolution displayed in Fig. S9(d-f), where DomiRank-based attacks tend to locally fragment the network's sub-structures in their attempt to dismantle the overall network. Finally, Fig. S9g shows that both of these methodologies are efficient at dismantling this toy network, slightly outperforming one another at different time steps, whilst being substantially better than other metrics at generating attacks to dismantle the network.

VII. LINK REMOVAL DURING ATTACKS

Our results have shown that attack strategies based on DomiRank centrality are more efficient in deteriorating the connectivity of the network in terms of the largest connected component than any other centrality-based attack. In this section (see Fig. S10), we also show that DomiRank-based attacks are able to remove links more efficiently than other attacks for synthetic and real-world networks, providing further evidence about the capacity of the DomiRank to highlight the nodes structurally important for the integrity of the network's connectivity.

The efficiency of DomiRank-based attacks in deteriorating network structural connectivity, both in terms of the largest connected component and sparsifying the number of connections, underlies the also outstanding capacity of this attack to severely impair the functionality of networks (see main text Fig. 6).

VIII. NETWORKS UNDERGOING RANDOM-RECOVERY

Complementing the results shown in Figure 5 in the main text, we show how a different recovery mechanism affects the evolution of the largest connected component under various attacks. Particularly we implement a random recovery mechanism, for which at every time step, a node is selected at random (with uniform probability) from the pool of the removed nodes. This selected node is recovered with probability p. Note that if a given node is recovered, it cannot be subject to any further attack. Our results using a random recovery mechanism are consistent with those shown in Figure 5 in the main text, where a sequential recovery mechanism was implemented. Notably, the high-σ DomiRank-based attack (Fig. S11) inflicts more enduring damage (i.e., longer time to recover the same relative size of

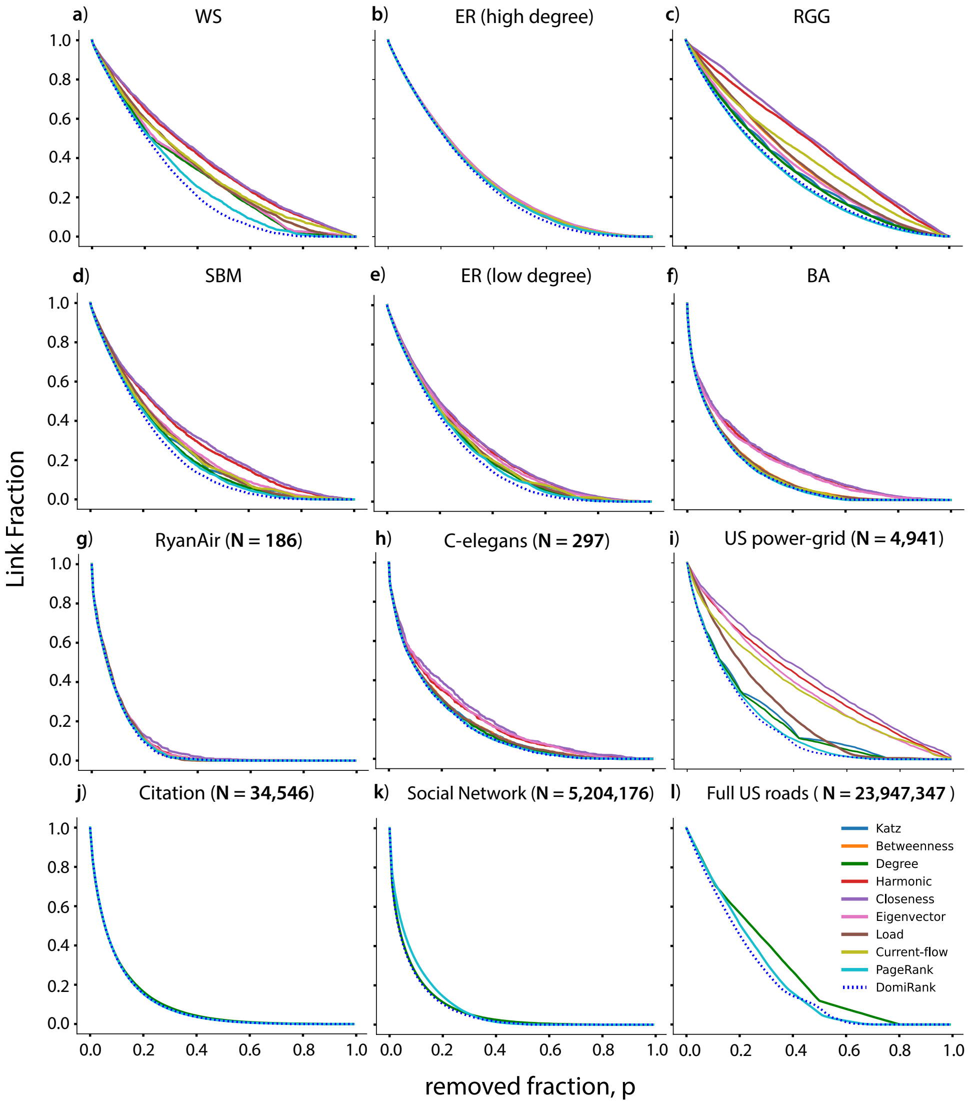

FIG. S10. **Link-removal on synthetic and real-world networks under centrality-based attacks.** Evolution of the remaining link fraction whilst undergoing sequential node removal according to descending scores of various centrality measures for different synthetic networks of size $N = 1000$: (a) Watts-Strogatz (WS; $\bar{k} = 4$), Erdős-Rényi (ER) with (b) high ($\bar{k} = 20$) and (e) low link density ($\bar{k} = 6$), (c) random geometric graph (RGG; $\bar{k} = 16$), (d) stochastic block model (SBM; $\bar{k} = 7$), and (f) Barábasi-Albert (BA; $\bar{k} = 6$). The performance of the attacks based on the different centrality metrics is also shown for different real networks: (g) hub-dominated transport network (airline connections, $\bar{k} = 16$), (h) neural network (worm, $\bar{k} = 29$), (i) spatial network (power-grid, $\bar{k} = 3$) , (j) citation network ($\bar{k} = 25$), (k) massive social network ($\bar{k} = 19$), and (l) massive spatial transport network (roads, $\bar{k} = 5$). Note that for panels j, k, and l, where massive networks are shown, only a few attack strategies are displayed due to the impossibility of computation of the rest.

the largest connected component) than the iterative betweenness when the network has a random recovery process, despite the fact that the iterative betweenness-based-attacks are superior in dismantling the structure of the network. Fig. S11a-d also showcases that when a network has a random recovery process, a low-σ DomiRank-centrality-based attack can result in an incrementally more rapid deterioration of the largest-connected-component than iterative betweenness. This fundamentally shows a key property of DomiRank-based attacks, i.e., the inherent trade-off between the efficiency of network dismantling and the endurance of the damage by modulating the parameter σ from low to high.

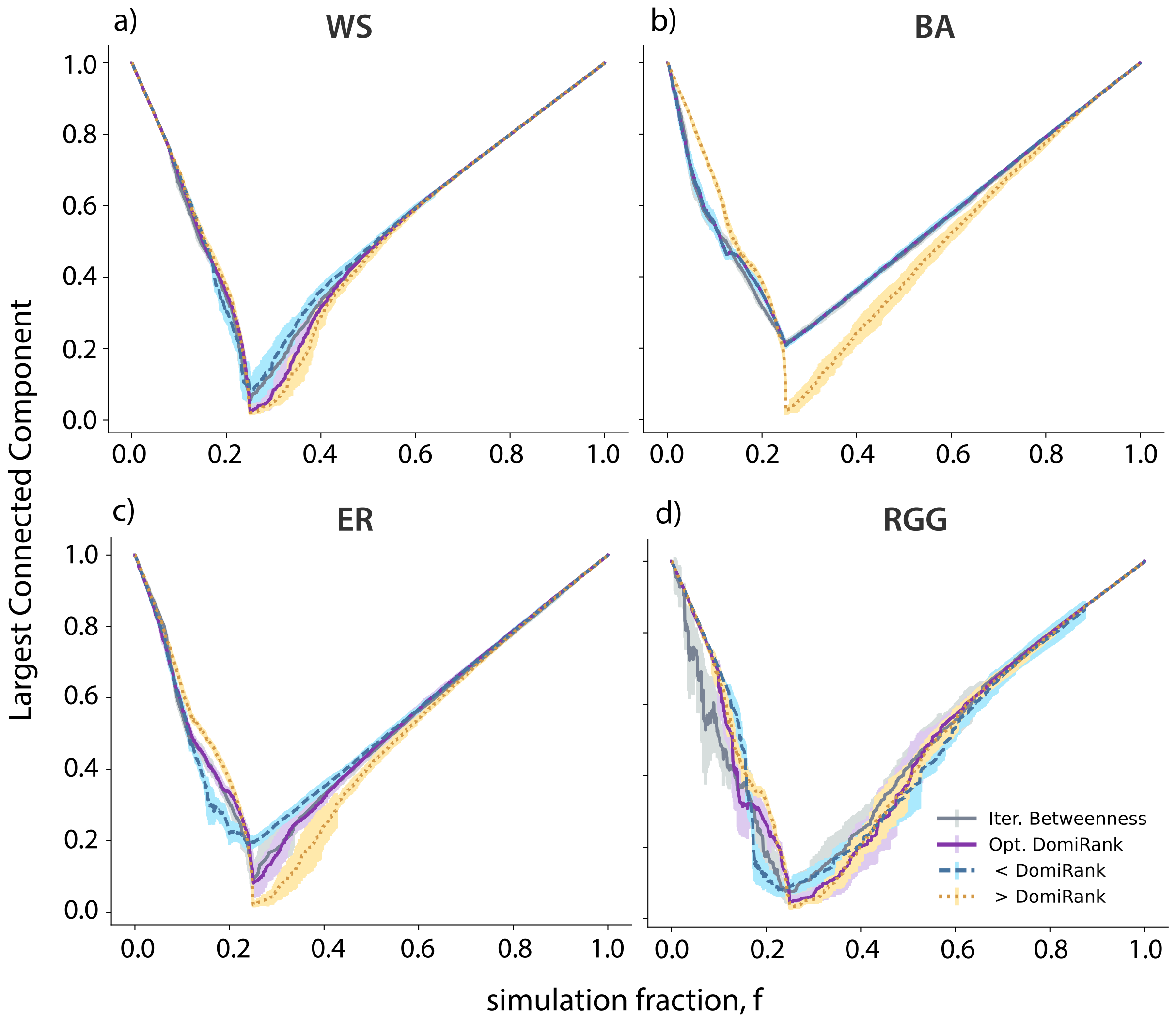

FIG. S11. **Evaluating the effect of random recovery during iterative betweenness-based and DomiRank attacks.** Evolution of the relative size of the largest connected component for various synthetic networks of size $N = 500$, namely (a) Watts-Strogatz (WS; $\bar{k} = 4$), (b) Barábasi-Albert (BA; $\bar{k} = 6$), (c) Erdős-Rényi (ER; $\bar{k} = 5$), and (d) random geometric graph (RGG; $\bar{k} = 7$), undergoing sequential node removal based on pre-computed DomiRank (optimal, low ($<$), and high ($>$) σ) and iterative betweenness, while a random node recovery process is ongoing; specifically, at each time step there is a probability of 0.25 to recover a random (already removed) node.

REFERENCES

[1] V. Colizza, A. Flammini, M. A. Serrano, and A. Vespignani, Detecting rich-club ordering in complex networks, Nature physics **2**, 110 (2006).

[2] J. J. McAuley, L. da Fontoura Costa, and T. S. Caetano, Rich-club phenomenon across complex network hierarchies, Applied Physics Letters **91** (2007).

[3] M. P. Van Den Heuvel and O. Sporns, Rich-club organization of the human connectome, Journal of Neuroscience **31**, 15775 (2011).

[4] J. Alstott, P. Panzarasa, M. Rubinov, E. T. Bullmore, and P. E. Vértes, A unifying framework for measuring weighted rich clubs, Scientific Reports **4**, 7258 (2014).

[5] D. J. Pearce, An improved algorithm for finding the strongly connected components of a directed graph, Victoria University, Wellington, NZ, Tech. Rep (2005).

[6] F. Morone and H. A. Makse, Influence maximization in complex networks through optimal percolation, Nature **524**, 65 (2015).

[7] F. Morone, B. Min, L. Bo, R. Mari, and H. A. Makse, Collective influence algorithm to find influencers via optimal percolation in massively large social media, Scientific reports **6**, 30062 (2016).

[8] S. Pei, F. Morone, and H. A. Makse, Theories for influencer identification in complex networks, in *Complex Spreading Phenomena in Social Systems: Influence and Contagion in Real-World Social Networks*, edited by S. Lehmann and Y.-Y. Ahn (Springer International Publishing, Cham, 2018) pp. 125–148.
